# Tissue Penetration of Antimicrobials in Intensive Care Unit Patients: A Systematic Review—Part II

**DOI:** 10.3390/antibiotics11091193

**Published:** 2022-09-03

**Authors:** Bruno Viaggi, Alice Cangialosi, Martin Langer, Carlo Olivieri, Andrea Gori, Alberto Corona, Stefano Finazzi, Antonello Di Paolo

**Affiliations:** 1 Department of Anesthesiology, Neuro-Intensive Care Unit, Careggi University Hospital, 50139 Florence, Italy; 2 Associazione GiViTI, c/o Istituto di Ricerche Farmacologiche Mario Negri IRCCS, 20156 Milan, Italy; 3 Department of Clinical and Experimental Medicine, University of Pisa, 56126 Pisa, Italy; 4 Anesthesia and Intensive Care, Sant’Andrea Hospital, ASL VC, 13100 Vercelli, Italy; 5 Infectious Diseases Unit, Foundation Istituto di Ricovero e Cura a Carattere Scientifico (IRCCS) Ca’ Granda Ospedale Maggiore Policlinico, 20122 Milan, Italy; 6 ICU and Accident & Emergency Department, ASST Valcamonica, 25043 Breno, Italy; 7 Istituto di Ricerche Farmacologiche Mario Negri IRCCS, 24020 Ranica, Italy

**Keywords:** antibacterial, penetration, critically ill patient, intensive care unit, fluoroquinolones, macrolides, tetracyclines, aminoglycosides, linezolid

## Abstract

In patients that are admitted to intensive care units (ICUs), the clinical outcome of severe infections depends on several factors, as well as the early administration of chemotherapies and comorbidities. Antimicrobials may be used in off-label regimens to maximize the probability of therapeutic concentrations within infected tissues and to prevent the selection of resistant clones. Interestingly, the literature clearly shows that the rate of tissue penetration is variable among antibacterial drugs, and the correlation between plasma and tissue concentrations may be inconstant. The present review harvests data about tissue penetration of antibacterial drugs in ICU patients, limiting the search to those drugs that mainly act as protein synthesis inhibitors and disrupting DNA structure and function. As expected, fluoroquinolones, macrolides, linezolid, and tigecycline have an excellent diffusion into epithelial lining fluid. That high penetration is fundamental for the therapy of ventilator and healthcare-associated pneumonia. Some drugs also display a high penetration rate within cerebrospinal fluid, while other agents diffuse into the skin and soft tissues. Further studies are needed to improve our knowledge about drug tissue penetration, especially in the presence of factors that may affect drug pharmacokinetics.

## 1. Introduction

Patients that are admitted to intensive care units (ICUs) have a variable risk of death depending on their health status, the severity of the disease, and the presence of comorbidities [1]. Notably, hospital admittance may be associated with the onset of new infections [2], as well as healthcare-associated pneumonia (HAP) and ventilator-associated pneumonia (VAP). Those infections often develop in ICU patients [3], and the COVID-19 pandemic has exacerbated that situation [4]. Furthermore, the rapid spreading of less sensitive bacterial strains or multidrug-resistant clones can worsen the clinical and microbiological outcomes of severely ill patients [5].

Some strategies can maximize the efficacy of antimicrobial drugs in ICU patients. For example, off-label doses of tigecycline improve the survival rate of patients, and they reduce the risk of mutant clone selection [6], despite treatment-associated toxicities increase [7]. Moreover, non-standard dosages can counteract the alterations of drug pharmacokinetics in ICU patients [8]. Sometimes those strategies fail to improve the outcome of chemotherapy. Ciprofloxacin in septic patients with augmented renal clearance may reach plasma (and tissue) concentrations that are lower than expected [9], the patients do not promptly recover from the infection, and the prolonged hospital stay may augment the risk of new-onset microbial diseases [10]. Furthermore, altered organ functions may influence the pharmacokinetics of other drugs in a variable manner [11,12].

Therapeutic drug monitoring (TDM) plays a pivotal role in the management of infections [13], so ICU bundles for antimicrobial stewardship include TDM protocols [14]. However, the correlation between plasma and tissue concentrations of drugs is variable and makes dose adjustment based on TDM findings challenging. Therefore, the tissue penetration rate of antimicrobials may guide the choice of the most appropriate chemotherapy.

The present work collected evidence about the tissue penetration rate of antimicrobials that were administered to ICU patients at the prescribed doses. The review evaluates drugs targeting protein synthesis, bacterial DNA, and folate pathways, as well as the main antitubercular drugs.

## 2. Results

**Figure 1:**
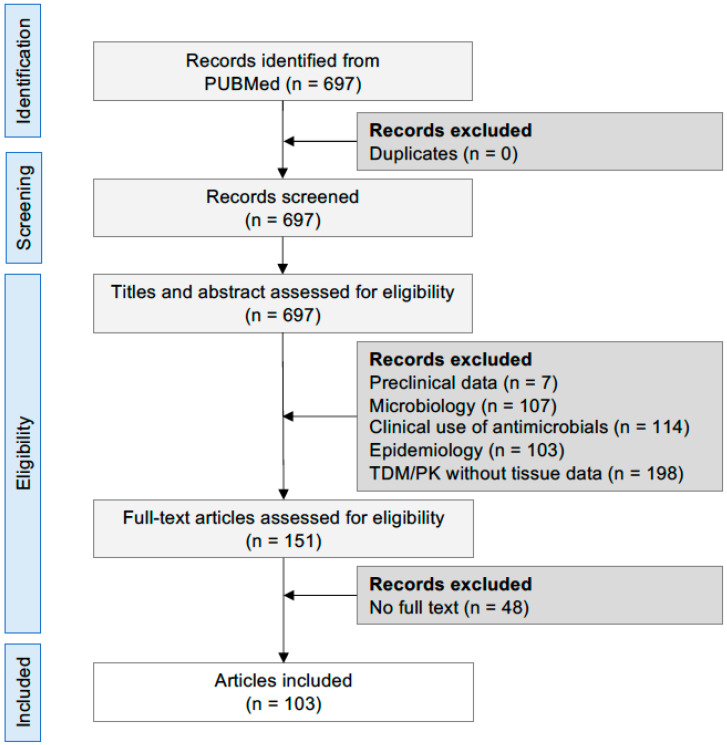
PRISMA flow diagram. Abbreviations: TDM, therapeutic drug monitoring; PK, pharmacokinetics.

The literature search found 697 articles about tissue penetration of antibacterial drugs in ICU patients, of which 103 were included based on the inclusion/exclusion criteria (Figure 1). The following sections describe the tissue penetration rate of antibacterial drugs inhibiting protein synthesis and DNA function, taking the corresponding plasma concentrations as a reference. The review also presents the administered doses and pharmacokinetic/pharmacodynamic (PK/PD) parameter values.

### 2.1. Fluoroquinolones

Fluoroquinolones are bactericidal drugs with a concentration-dependent killing. The ratio between the area under the curve (AUC) of plasma concentrations and the MIC (AUC/MIC) is predictive of drug efficacy with threshold values ≥ 100 [15,16]. Even the maximum plasma concentration (C_max_)/MIC ratio may predict treatment efficacy, and the threshold value is approximately 10.

Ciprofloxacin penetration in **brain** tissue was evaluated in 14 patients who underwent surgical excision of tumors [17]. A total of 60 minutes after a single dose of 200 mg i.v., the mean tissue/plasma ratio of ciprofloxacin was 0.88X (SD, 0.99X) in the brain (Table 1), a value that was lower than that which was calculated for **subcutaneous fat** (1.34–1.40X) and **dura mater** (2.26X), but higher than that which was obtained in **skull bone** (0.68–0.75X).

Ciprofloxacin has a longer half-life (t_1/2_) in liquor than in plasma [32], and its penetration rate into the cerebrospinal fluid (**CSF**) depends on the meningeal inflammation. Indeed, among 23 patients with purulent meningitis and 3 with ventriculitis, ciprofloxacin 0.2 g every 24 h (q24h) achieved tissue/plasma ratios of 0.26–1.59X and 0.14–0.78X in the presence or absence of meningeal inflammation, respectively [33]. Of note, the actual mean maximum CSF concentrations ranged between 0.49 and 0.56 mg/L 2–4 h after dosing. Furthermore, in 4 out of the 23 patients, multiple ventricular CSF samples were collected by external ventricular drainage (EVD), and the peak concentrations were 0.25–0.45 mg/L 2–6 h post-dosing. In another study, ciprofloxacin 0.2 g every 12 h (q12h) achieved CSF concentrations of 0.073–0.106 mg/L and 0.089–0.260 mg/L in the presence and absence of meningeal inflammation, respectively [34]. Although ciprofloxacin has a lower penetration rate in CSF with respect to other fluoroquinolones, those concentrations exceeded the MIC values of most Gram-aerobic bacilli. Some studies investigated higher dosage regimens. In particular, ciprofloxacin 0.4 g every 8 h (q8h) led to “hypothesized” CSF concentrations of approximately 0.9 mg/L [35], which could be more effective. Furthermore, the mean CSF/plasma AUC ratio was 0.26X in 16 patients that were affected by tuberculous meningitis who received ciprofloxacin 0.75 g q12h resulted in a mean CSF/plasma AUC ratio of 0.26X [21].

A single i.v. dose of ofloxacin 0.2 g in 10 cancer patients yielded peak CSF concentrations of 0.4–1.0 mg/L 2–4 h post-dose, with concentrations >0.1 mg/L for 24 h [36]. Another study demonstrated that doses of 0.2 g q12h diffused into CSF with a mean AUC ratio of 0.76X and 0.73X in 22 patients with meningitis and ventriculitis, respectively [31]. When the meningeal inflammation resolved, the mean CSF/plasma ratio ranged between 0.30 and 1.34X. As reported for ciprofloxacin, the mean terminal half-life (t_1/2_) in CSF (10.2 h) was longer than in plasma (7.1 h). Both clinical trials adopted a lumbar puncture (LPD) to collect CSF. On the contrary, an EVD was used to harvest CSF samples in six patients with occlusive hydrocephalus [37]. Interestingly, ofloxacin 0.4 g achieved a mean CSF/plasma ratio of 0.65X (range 0.59–0.81X), and the peak concentration in CSF ranged from 1.0 up to 2.85 mg/L. Therefore, the authors concluded that high doses of ofloxacin could be effective only against the most susceptible bacterial strains (i.e., MIC ≤ 0.1 mg/L) according to a C_max_/MIC target value of ≥10.

A similar CSF penetration rate was calculated for levofloxacin 0.5 g q12h in 10 patients with EVD [38], because the AUC and C_max_ ratios accounted for 0.71X and 0.47X, respectively. According to the PK/PD thresholds for C_max_/MIC (12.2) and AUC/MIC (125 h), MIC values ≤ 0.5 mg/L could predict a positive outcome of chemotherapy. At the same dose of 0.5 g q12h, levofloxacin achieved a mean CSF/plasma AUC ratio of 0.74X (range, 0.58–1.03X) in 15 patients with tuberculous meningitis [21]. In that study, the CSF was sampled through an LPD.

Moxifloxacin 0.4 g as a single i.v. dose achieved mean maximum concentrations in CSF of 4.07 mg/L 4–6 h post-dose [39]. In tuberculous meningitis, moxifloxacin 0.4–0.8 g q24h resulted in median CSF/plasma AUC ratios of 0.82–0.71X [40]. The AUC ratio was in the range of 0.85–1.75X when unbound CSF and plasma concentrations were considered. Finally, PK/PD analysis in patients with tuberculous meningitis revealed that optimal outcomes (i.e., survival, death/disability, time to death) were better related to AUC/MIC ratios in CSF [21,41].

Overall, fluoroquinolones have an optimal diffusion into the respiratory tract, especially in epithelial lining fluid (**ELF**) and **alveolar cells**, and they are prescribed to treat severe infections of the lower respiratory tract (LRTI) [19]. Ciprofloxacin 0.2 g i.v. achieved tissue/plasma ratios that were higher than 2X for the entire sampling time interval (i.e., 5 h post-dose) in **bronchial mucosa**, **lung parenchyma**, and **pleura** in 20 cancer patients [42]. In particular, the mean concentrations in these tissues were always greater than 1.3, 2.1, and 0.9 mg/L, respectively. Higher doses of ciprofloxacin (i.e., 0.4 g q8h) achieved a mean **bronchial secretion**/plasma AUC ratio of 1.16X in 25 mechanically ventilated patients that were suffering from severe chronic obstructive pulmonary disease [23]. The data showed that the C_max_/MIC ratio was ≥10 in all patients for MIC values ≤ 0.5 mg/L, but drug exposure could be inadequate for higher MIC values (i.e., >0.5 mg/L).

Further studies investigated tissue penetration of fluoroquinolones after oral doses. Single doses of ciprofloxacin 0.5 g and levofloxacin 0.5 g accumulated in **alveolar macrophages** (AM) up to 10X [43,44]. In healthy volunteers (HV) at the steady state, oral levofloxacin 0.5–0.75 g achieved tissue/plasma ratios that were greater than 2X in ELF and 10X in AM 24 h post-dose [45]. The different penetration rates between ciprofloxacin and levofloxacin could depend on their bioavailability (78% and 100%, respectively) [46]. Furthermore, the high diffusion of levofloxacin 0.5 g i.v. q12h or q24h in LRT has been confirmed in 24 ICU patients with community-acquired pneumonia (CAP) [24]. In particular, the median AUC values in **ELF** were 151 (range, 137–174) and 208 (range, 203–236) hxmg/L for the q12h and the q24h schedule, respectively, with actual AUC/MIC values greater than 172 h in 23 out of 24 patients. Of note, those values in the ELF exceeded the PK/PD thresholds that were predictive of outcome for MIC >1 mg/L. Finally, 15 uninfected cancer patients and 18 patients with acute exacerbations of chronic bronchitis received 5 oral doses of levofloxacin 0.75 g q24h [47]. Notably, tissue inflammation caused an increased ELF volume that significantly diminished levofloxacin concentrations. In particular, nearly 60% of patients with chronic bronchitis did not reach an ELF/unbound plasma ratio of 1X. For this reason, the authors suggested a careful evaluation of levofloxacin tissue penetration in the presence of inflammation.

A single oral dose of moxifloxacin 0.4 g in 17 HV achieved tissue/serum ratios greater than 5X in ELF and 1.5X in bronchial mucosa up to 24 h post dose [48], while the ratio ranged from 18X up to 70X in AM [49]. In lung parenchyma, the tissue/plasma ratio was always higher than 2X up to 36 h after multiple i.v. or oral doses of 0.4 g q24h. As previously discussed for levofloxacin, moxifloxacin displays an altered diffusion in inflamed tissues. Indeed, multiple i.v. doses of 0.4 g q24h achieved a mean penetration rate in the bronchial secretion of 0.99X (range, 0.35–1.53X) and 0.80X (range, 0.17–1.37X) of patients that were admitted to a general ward and ICU, respectively [29]. Although that difference was not statistically significant, the pharmacokinetic variability associated with the infection could impair the attainment of PK/PD target values.

Lastly, patients that were affected by chronic bronchitis received single and multiple oral doses of ofloxacin 0.2 g q12h [50]. The penetration rate in bronchial mucosa was at least 1X and up to 9X 2 h after the last dose. Interestingly, the high penetration of oral ofloxacin 0.2 g q12h was confirmed in ELF (4.9X) and AC (>5X), whereas the penetration rate did not differ in healthy and pathological lung tissues [51]. Therefore, the penetration rate of ofloxacin allowed a long-lasting antimicrobial activity against most potential respiratory bacteria.

Fluoroquinolones do attain high tissue/plasma ratios also in **bone**. A single dose of ciprofloxacin 0.2 g did achieve tissue/plasma ratios of 0.44–0.75X in skull bone [17], whereas multiple doses yielded a higher penetration rate. Indeed, ciprofloxacin 0.75 g q12h followed by an i.v. infusion of 0.4 g had mean peak concentrations of 8.8 mg/L 1–3 h after the last dose in the sternal bone [52]. Those bone concentrations corresponded to tissue/plasma ratios of 2X or greater. Furthermore, a single i.v. levofloxacin 0.5 g i.v. achieved mean tissue/plasma ratios of 1.2X, 1.0X, and 0.5X in synovia, cancellous, and cortical bone, respectively [53]. Of note, the tissue concentrations were higher than the breakpoint values for susceptible bacteria. In 16 orthopedic patients with severe forelimb ischemia, levofloxacin 0.5 g q24h achieved a bone/plasma ratio of 0.28–0.44X 1 h after the last dose [26], showing that drug penetration was not influenced by the degree of ischemia. Finally, sternal bone concentrations of ofloxacin 0.2 g q12h were stable up to 10 h after the last dose, with actual mean values of 2.56–2.79 μg/g [54].

The diffusion of fluoroquinolones into the interstitial fluid (ISF) of subcutis and muscle of has not been investigated in ICU patients. On the contrary, some interesting data have been obtained in HV using the microdialysis technique. A single i.v. dose of ciprofloxacin 0.4 g did generate a mean tissue/plasma AUC ratio of 0.68X and 0.38X in **muscle** and **subcutis**, respectively [55]. Furthermore, the mean AUC ratios were 0.93X, 0.46X, and 1.46X in **capillary**, **saliva**, and **blister fluid**, respectively. The highest value in blister fluid was likely due to the greater penetration of ciprofloxacin into inflamed tissues. Of note, the mean actual C_max_ values accounted for 4.34, 1.24, 1.18, and 1.40 mg/L in plasma, muscle, subcutis, and blister, respectively. A single oral dose of ciprofloxacin 0.5 g resulted in mean tissue/plasma ratios ranging from 0.55X [56] up to 1.44X [55].

Levofloxacin has a greater tissue penetration rate than ciprofloxacin. In 21 orthopedic patients, a single i.v. dose of levofloxacin 0.5 g had median concentrations of 7.95 mg/L, 5.14 μg/g, and 7.94 μg/g in plasma, cancellous bone, and muscle, respectively, 40–210 min after dosing [57]. More interestingly, the drug displayed an increased penetration into inflamed tissues. Indeed, levofloxacin achieved effective concentrations in granulomatous tissue (11.45 μg/g), wounds (19.51 μg/g), and skin (19.89 μg/g) [57]. Similar findings were obtained in skin samples of 11 HV receiving oral levofloxacin 0.75 g q24h [58]. The tissue/plasma ratio increased over time from 1.47X up to 4.68X, with a mean AUC ratio of 1.97X. The mean actual C_max_ value was 11.87 μg/g of tissue 6 h after the last dose, for a C_max_ tissue/plasma ratio of 1.37X. Finally, 10 diabetic patients with foot ulcers were treated with oral levofloxacin 0.5 g q24h [59]. In agreement with other studies, the median levofloxacin concentrations were 9.84 mg/kg in wound tissues and 2.42 mg/L in plasma.

After a single i.v. or oral doses of moxifloxacin 0.4 g in 12 HV, the penetration rate was 0.55X in muscle and 0.38X in subcutaneous adipose tissue [60]. Notably, those ratios increased up to 0.86X and 0.81X, respectively, when the unbound fraction of plasma concentrations was considered. As observed for ciprofloxacin [55], the penetration of moxifloxacin in some tissues (i.e., muscle and adipose tissue) achieved the equilibrium with plasma earlier than in other compartments (i.e., blister fluid). The measured C_max_ values were 3.7, 1.2, 1.0, and 1.7 mg/L (or μg/g) in plasma, muscle, subcutis, and blister, respectively. In 8 HV a single dose of moxifloxacin 0.4 g had a high penetration (approximately 1X) into the inflamed tissues regardless of the oral or i.v. route of administration, with mean time-to-peak (T_max_) values of 2.43 h [61]. Therefore, moxifloxacin achieved bactericidal concentrations in the ISF. Similar results (1.03–1.20X) were observed for a single oral dose of ofloxacin 0.3 g in 8 HV [62].

Overall, fluoroquinolones have a high diffusion into peripheral compartments, and the penetration rate could be partly influenced by the inflammation [63] and illness severity. For example, a significant correlation was found between the volume of distribution of levofloxacin and the sickness severity [64]. On the contrary, minor differences in drug PK were observed in patients with severe sepsis or intra-abdominal infections [12]. Those findings support an appropriate choice of antimicrobial chemotherapy that may decrease the risk of selecting resistant bacterial clones in ICU patients [65].

### 2.2. Aminoglycosides

The concentration-dependent killing of aminoglycosides is predicted by both C_max_/MIC and AUC/MIC ratios, with efficacy threshold values accounting for ≥8 and ≥30–50, respectively [66]. More recently, Bland and colleagues suggest that higher AUC/MIC target values (i.e., 80–100) should be considered in ICU patients, especially in the presence of severe illness, immuno-compromised hosts, and high bacterial burden [66].

Data about **CSF** penetration of aminoglycosides are available in newborns and children, using an LPD for CSF sampling. In 44 neonates who received amikacin (15.5–20 mg/kg every 42–24 h), the CSF/plasma AUC ratio was approximately 0.1X (Table 2) [67]. In agreement with those results, the CSF/plasma C_max_ ratio accounted for 0.08X in 16 children (age, 7 months–8 years) [68]. Therefore, the CSF penetration of amikacin is similar to that of beta-lactams [22].

In **bronchial secretion**, amikacin 1 g q24h and 0.5 g q12h achieved mean tissue/serum AUC ratios of 0.46X and 0.66X on day 1 and 0.57X and 0.81X on day 3, respectively [69]. Of note, the once-daily schedule resulted in higher mean C_max_/MIC values, with peak concentrations of 13.6 mg/L 3 h post-dosing. Amikacin penetration in ELF allowed bactericidal concentrations against less sensitive bacterial strains. However, another study measured amikacin **ELF**/plasma penetration rate 2 h post-dose in 8 VAP patients (mean adjusted body weight, 70 kg) who received a mean dose of 20 mg/kg q24h [70]. The median ELF concentration was 3.6 mg/L (interquartile range, IQR, 2.1–13.4 mg/L) with a mean (median) ELF/plasma ratio of 0.10X (0.07X) and 0.18X (0.09X) 1 and 2 h after dose, respectively. That penetration rate was too low to treat infections that were sustained by the less sensitive bacterial strains.

In the case of tobramycin 7–10 mg/kg, work by Boselli and coworkers estimated the ELF distribution of the drug in 8 VAP patients [71]. The samples (serum and bronchoalveolar lavage, BAL) were collected 30 min after the end of the 0.5 h infusion, and the BAL/serum ratio was 0.12X. The authors considered the penetration rate of tobramycin 7–10 mg/kg ineffective in treating LRTI. It is interesting to note that other studies found a higher tobramycin diffusion into ELF. A total of 16 pneumonia patients received tobramycin q8h at doses that were optimized to achieve peaks and through plasma concentrations of 8 mg/L and <2 mg/L, respectively [72]. The ELF/serum ratio was 0.30X-1.56X up to 8 h after dosing, while the mean ELF concentrations ranged from 2.33 up to 0.77 mg/L. Therefore, high tobramycin doses were required to obtain effective ELF concentrations. An ELF/serum ratio of 1.40X-1.60X was obtained in 10 ICU patients who received tobramycin 0.3 g by intramuscular injection [73]. Finally, gentamycin 0.24 g q24h had an ELF penetration rate that was similar to tobramycin. Indeed, the ELF/serum ratio accounted for 0.30–1.14X in 24 VAP patients [74].

It is worth noting that changes in the patients’ clinical conditions may significantly influence aminoglycoside pharmacokinetics. For example, gentamycin pharmacokinetics changed in 40 ICU patients that were affected by severe Gram-infections [75]. Moreover, burn injuries altered the pharmacokinetics of amikacin [76] and tobramycin [77]. Those changes require higher individualized doses [78,79,80,81] and, more importantly, may influence the tissue penetration of drugs.

**Table 2:** The tissue/plasma ratio values for aminoglycosides, clarithromycin, and azithromycin. For each drug, the different daily doses that were administered to ICU patients are listed in the table.

Drugs	Gentamycin	Amikacin	Tobramycin	Clarithromycin	Azithromycin
Daily doses	−5 mg/kg i.v. −240 mg	−15 mg/kg i.v. −7.5 mg/kg q12h −20 mg/kg	−5 mg/kg i.v. −300 mg i.m.	−500 mg q12h	−500 mg/day × 3 days
CSF		0.1X			>50X ^A^
Lung		0.4X			>60X
ELF	0.3–1.14X ^B^	0.09X	0.12X–1.6X		>7X/>40X ^C^
Bronchial secretion		0.46–0.57X			
Bone	0.17–0.5X		0.5X	0.7X	
Synovial fluid	>1X	>1X			
Skin					>1X
**References**	[27,28,74]	[22,27,28,67,69,70]	[27,71]	[20]	[82,83,84,85,86]

**Notes**: ^A^, brain; ^B^, minimum-maximum values across the selected references; ^C^, alveolar macrophages. **Abbreviations**: CSF, cerebrospinal fluid; ELF, epithelial lining fluid; q12h, every 12 h.

### 2.3. Macrolides and Azalides

Macrolides and azalides have a concentration-dependent bacterial killing that can be predicted by the AUC/MIC and C_max_/MIC ratios [15], with target values of >30 and >8, respectively.

Azithromycin 0.5 g penetrates the **brain** at tissue/plasma ratios ≥50X up to 96 h after the administration of a single oral dose [82] (Table 2).

Several studies described the high azithromycin penetration in LRT. In particular, **ELF**/plasma AUC ratios after single doses of 0.5 g and 1.0 g were 2.96X and 5.27X, respectively. Higher AUC ratios were observed in the **lung parenchyma** at both dose levels (i.e., 64.35X and 97.73X, respectively) [84]. In 24 cancer patients who received a single dose of azithromycin 0.5 g, the tissue/plasma ratio was >40X in the lung parenchyma and **AM [87]**. Those results confirmed the high penetration of azithromycin into the infection sites in experimental models of pneumonia [88] and drug accumulation within white blood cells [86]. Furthermore, single and repeated doses of azithromycin had a preferential distribution in AM (>100X), with a progressive increase up to 24–120 h after the last dose [89,90,91].

In agreement with those results, clarithromycin has excellent penetration in the **lungs**. In 10 patients, oral clarithromycin 0.5 g q12h reached high tissue/serum ratios in the bronchial mucosa (>4X), ELF (>4X), and AM (>100X), approximately 4.25 h after dosing [92]. In HV who received single or multiple doses, the ELF penetration rate of oral clarithromycin 0.5 g was >10X 4–6 h after dosing [89,90,93], while the AM/serum ratio was >100X, with concentrations detectable up to 24 h after a single dose of 0.5 g [89]. Clarithromycin 0.2 g did generate BAL/serum AUC ratios of 3.5X in 5 HV, with T_max_ values of 5.2 h in BAL [94]. Interestingly, the presence (3.8–7.1X) or absence (3.0–17.8X) of *Mycobacterium avium* complex (MAC) lesions did not significantly influence clarithromycin penetration into BAL [95]. Therefore, doses of 0.8 g/day are adequate to impede the intrapulmonary spreading of MAC [95].

Erythromycin 0.25 g q6h p.o. was detectable in ELF (mean concentration, 0.8 mg/L 4 h post-dosing) and in AC (0.1–0.8 mg/L 8–12 h post-dosing) [93].

A POP/PK study developed a PK model to predict tissue distribution of azithromycin 0.5 g q24h for 3 days in 6 HV [96]. Azithromycin had a high volume of distribution and it accumulated within polymorphonuclear **leukocytes**. In particular, intracellular concentrations were higher than the MIC values of pathogens that were responsible for **skin** infections (i.e., *S. aureus*, MIC 2 mg/L). Therefore, azithromycin was effective against skin infections despite the low unbound concentrations of the drug in both **muscle** and **subcutis** [97]. Another study enrolling six HV confirmed those findings [86], with penetration ratios in leukocytes varying from 145X on day 1 up to 1800X 7 days after the last dose [86].

A limited number of studies investigated **bone** penetration of macrolides and azalides. Azithromycin seems to have a high penetration rate in bone (up to 6.3X) [20].

### 2.4. Other Antibacterial Drugs

#### 2.4.1. Linezolid

A target AUC/MIC ratio of 80–120 and a T > MIC value ≥ 85% are predictive of linezolid efficacy [98]. Both parameters may forecast antimicrobial activity in the presence of factors influencing linezolid pharmacokinetics [11,99].

Linezolid does penetrate the **CSF**, where it rapidly achieves tissue/plasma ratios equal to 0.7–0.9X regardless of the meningeal inflammation (Table 3) [22,100,101,102,103]. In particular, the CSF/plasma ratio was approaching 1X 2 h after dosing [102], while CSF minimum concentrations exceeded the MIC values for sensitive pathogens. However, the large variability in CSF concentrations may explain why PK/PD parameters T > MIC or AUC/MIC were not higher than the recommended threshold values in some patients [101,103,104,105]. Furthermore, bacterial species that were less sensitive to linezolid (i.e., MIC ≥4 mg/L) which reduced the probability of target attainment [102,105]. The findings suggest higher doses of linezolid are required (i.e., 0.6 g q8h).

Linezolid promptly diffused into the **ELF** that was collected from 16 VAP patients [106]. The ELF/plasma ratio was 1X for both peak and through concentrations, being effective against most bacterial strains with MIC values of 2–4 mg/L. In 12 VAP patients, the administration of linezolid according to a loading dose (0.6 g) followed by a continuous infusion (1.2 g/day) was associated with a median ELF/plasma ratio of 1X (IQR, 0.8–1.1X) [107]. Notably, continuous infusions of linezolid may reduce the interindividual variability in ELF concentrations [106]. Indeed, in 22 critically obese patients, a loading dose (0.6 g) followed by a continuous infusion (1.2 g) produced an ELF/plasma ratio of 1.06X, which was higher than that (0.80X) which was obtained with standard treatment (i.e., 0.6 g q12h) [108]. However, the alternative regimen could have a reduced efficacy in the presence of bacterial strains with MIC values ≥ 4 mg/L.

Linezolid yielded tissue/plasma ratios of 0.23X in the **bone** [20], and higher values (0.4–0.75X) were obtained in orthopedic patients approximately 0.5–1.5 h after dosing. Moreover, in nine patients with spinal TBC, a single oral dose of 0.6 g led to a median **pathological bone**/plasma ratio of 0.48X (range, 0.30–0.67X) [109]. Oral linezolid 0.6 g q12h had a mean tissue/serum ratio of 0.46X (range, 0.18–0.71X) 1–12 h after dosing in six orthopedic patients with diabetic foot infections [110].

The diffusion of linezolid into the **skin** achieved therapeutic concentrations [111] without being influenced by blood perfusion and ischemia [26]. In 12 patients with sepsis or septic shock, the microdialysis sampling allowed the measurement of unbound ISF concentrations of linezolid [112]. The median tissue/serum AUC ratios accounted for 0.9X (range, 0.2–1.2X) and 1.0X (0.2–1.4X) in the subcutis and the muscle, respectively [112]. However, the *f*T > MIC value of subcutis and muscle was below 40% in four and two out of nine patients, respectively, suggesting that “*the large range of the calculated data was remarkable*”. Furthermore, the ISF/serum ratios of approximately 1X were obtained in patients with septic shock (n = 16) or severe sepsis (n = 8), regardless of the illness severity. Those ratios were similar to those that were calculated in HV [113].

The variable activity of the ABCB1 transmembrane transporter and drug-drug interactions could explain the PK variability of linezolid among patients [117,118]. The role of these factors in ICU patients is still under evaluation.

#### 2.4.2. Tetracyclines and Glycilglycine

Tetracyclines and tigecycline have concentration-dependent killing, and the AUC/MIC parameter predicts their efficacy. In particular, the AUC/MIC target values for tigecycline ranged between ≥1 (for VAP and bone infections) up to ≥18 (for complicated skin and skin-structures infections) [119].

Doxycycline displays a reduced **CSF** penetration rate (approximately 0.2X) [114], while **ISF** penetration accounted for approximately 0.5X [115].

Similar mean CSF/plasma AUC ratios (0.1X) were obtained for tigecycline [116]. In patients that were undergoing elective surgery, a single i.v. dose of 0.1 g extensively distributed in the **lung** parenchyma (tissue/serum ratios, 2.4–11.2X) and **colon** (2.3–11.9X) up to 24 h after dosing [116]. In HV receiving standard doses, tigecycline achieved mean tissue/serum AUC ratios of 1.7X in ELF and 20.8X in AC [120]. That high penetration in LRT sustains the use of tigecycline as second-line, long-lasting chemotherapy for HAP and VAP [121].

Of note, tigecycline doses are often doubled to increase the probability of a cure for ICU patients [122]. However, those high doses did not decrease the pharmacokinetic variability among 37 adult ICU patients [123]. Therefore, Borsuk-De Moor and colleagues suggested an individual dose adjustment. Finally, the unbound plasma fraction of tigecycline decreases at higher concentrations [124].

High tissue/serum ratios were measured in the gallbladder (>34X) and bile (>600X) thanks to the biliary excretion of tigecycline [125], while the penetration rates were 0.4–2X in the **bone** and 0.6–0.9X in **synovial fluid** after a single dose of 100 mg [116]. Multiple doses of tigecycline had a high penetration rate into healthy and infected tissues, with an unbound **ISF**/serum AUC ratio of 1X [126]. Overall, those data confirm the penetration of tigecycline in the LRT and soft tissues, especially after multiple doses.

#### 2.4.3. Clindamycin

The AUC/MIC predicts the antibacterial activity of clindamycin [127]. Of note, bioassay techniques were used to measure drug concentrations in all the studies except for one [26].

In 10 AIDS patients, clindamycin 1.2 g achieved tissue/plasma ratios <0.02X in **CSF** that was collected by LPD 1.5 or 2.5 h after dosing [128].

After a single i.v. dose, clindamycin 0.6 g rapidly diffused in the **muscle** and **oral mucosa** of 31 patients who underwent maxillofacial surgery [129], and the drug was detectable up to 8 h post-dosing. Lower concentrations were measured in **bone**, **skin**, and **adipose tissues** [129], but they were higher than the MIC values of the most common pathogens. On the contrary, in 29 subjects with decubitus ulcers, the tissue/plasma ratios were 1X for both bone and skin 0.5–1.5 h after dosing [130]. In lower limb ischemia, the different rates of perfusion influenced the penetration of clindamycin 0.6 g q8h in muscle (0.4–0.5X), bone (0.2–0.3X), and skin (0.2–0.4X) [26].

Finally, in 15 children, clindamycin 10 mg/kg (≈0.3 g) did penetrate inflamed appendices and **peritoneal fluid**, achieving concentrations that were approximately equal to those that were measured in plasma [131].

#### 2.4.4. Metronidazole

The AUC/MIC ratio may predict the antibacterial activity of metronidazole, with threshold values ≥ 70 for *B. fragilis* [132,133].

Metronidazole diffuses into **CSF** [134]. Indeed, metronidazole 0.5 g q8h reached a mean CSF/unbound serum AUC ratio of 0.87X in four traumatic patients with an EVD [135]. Notably, metronidazole 0.5 g q8h reached a mean ISF/unbound serum AUC ratio of 1.02X in the **brain** parenchyma [136].

Tissue concentrations of metronidazole 1.5 g were 3.3–41.7 μg/g in the **peritoneum** and 6.7–43.1 μg/g in the **colon** wall up to 36 h after dosing [137]. The concomitant mean plasma concentrations decreased from 39.9 ± 17.1 mg/L at the end of the infusion up to 2.6 ± 1.1 mg/L 36 h after dosing. Those findings suggested that metronidazole could exert an effective prophylactic activity in abdominal surgery.

A single i.v. dose of metronidazole 0.5 g yielded a mean ISF/serum AUC ratio of 0.88X in the **muscle** of six septic patients [138]. A following simulated in vitro kinetics demonstrated a rapid bactericidal effect against two *B. fragilis* strains (MIC values, 0.125 and 1 mg/L). In the **skin**, the mean ISF/plasma AUC ratio was 0.67X after the oral administration of metronidazole 2 g [139]. In six rheumatology inpatients, the **synovial fluid**/serum ratio of oral metronidazole 0.4 g q8h was 1X 3 h after the first dose [140]. Interestingly, synovial concentrations remained higher than 3.6 mg/L up to 36 h after dosing, and they were similar to the breakpoint of *B. fragilis* (i.e., 4 mg/L).

#### 2.4.5. Rifampin, Isoniazid, and Chloramphenicol

The AUC/MIC ratio is associated with the antibacterial activity of rifampin, isoniazid, and pyrazinamide [141,142]. Moreover, the T > MIC index predicts the antibacterial effects of chloramphenicol [143].

All antitubercular agents penetrate CSF except for rifampin. Indeed, rifampin diffused into **CSF** with a penetration rate of 0.22–0.3X [22]. In 237 patients with tuberculous meningitis, the median CSF/plasma AUC ratio accounted for 0.07X regardless of the rifampin dose (10 or 20 mg/kg/day) [144]. Consequently, the probability of attaining AUC_0–24 h_/MIC values > 297 in the CSF was very low for *M. tuberculosis* strains with MIC ≥ 0.5 mg/L [145]. In agreement with those results, another study enrolling 30 patients with tuberculosis demonstrated a low serum-to-CSF passage at intensified doses [146]. Indeed, the highest CSF concentration of rifampin correlated with plasma C_max_ value, but the CSF/plasma C_max_ ratio was always <0.1X [147]. Moreover, all of the patients except two had rifampin CSF concentrations that were lower than the MIC values of susceptible bacteria.

On the contrary, the penetration ratio of isoniazide within **CSF** was equal to 1X in 237 patients with meningeal tuberculosis (TBC) [144]. Chloramphenicol had CSF/plasma ratios of 0.6–0.7X [22,148].

The penetration of rifampin into the **LRT** has been evaluated by bioassay in 15 patients who received a single oral dose of 0.6 g [149]. The mean tissue/plasma ratios were 0.34X, 0.51X, and 16.26X in **ELF**, **bronchial mucosa**, and **AM**, respectively. In 40 patients (with or without AIDS) that were treated with rifampin 0.6 g q12h, the mean ELF/serum and AM/serum ratios were 0.2X and 0.9–1.5X, respectively, 4 h post-dosing [150]. Although the penetration rate of rifampin within AM, other authors suggested higher rifampin doses to attain the desired antitubercular effect [151].

In 80 patients with AIDS or not, isoniazid 0.3 g q24h generated higher mean **ELF**/serum ratios in slow acetylators (3.2X) than in fast ones (1.2X) [152]. Moreover, the mean **AM**/serum ratio was 2.1X in the same patients.

Finally, a study in 14 patients that were affected by spine TBC, rifampin 10 mg/kg/day yielded **bone**/plasma ratios of 0.54–0.66X 2–3 h after dosing [153]. Interestingly, drug penetration into the infective foci was significantly lower (0.06–0.08X) than in healthy tissue, and rifampin was undetectable in the presence of a sclerotic wall around the foci.

#### 2.4.6. Cotrimoxazole (Trimethoprim-Sulfamethoxazole, TMP-SMZ)

The AUC/MIC parameter predicts the antibacterial activity of both TMP and SMZ [154].

Cotrimoxazole remains an effective drug to control and treat infections that are caused by MDR strains as in the case of MRSA [155].

The penetration of TMP-SMZ 0.005/0.025 g/kg i.v. has been evaluated in nine patients with the vertebral disease [156], showing that the **CSF**/serum AUC ratio was 0.18X for TMP and 0.12X for SMZ. The mean actual AUC values of TMP (32.6 hxmg/L) and SMZ (1160 hxmg/L) ensured the attainment of *f*AUC/MIC values > 25 [157]. Of note, TMP had a CSF T_max_ value (1 h) that was lower than those that were observed for SMZ (8 h).

After multiple doses of cotrimoxazole 0.16/0.8 g, the TMP **synovial fluid**/plasma ratio was approaching 1X 3 h post-dosing, while for SMZ the ratio was about 0.75X 6 h post-dosing [158]. Those data reflected the faster tissue diffusion of TMP in comparison with SMZ. A total of 12 patients with diabetic foot infection received standard (0.16 g/0.8 g) or high oral doses (0.32/1.6 g) q12h [110]. The findings showed that the penetration rate of TMP (1.2X, range, 0.4–2.2X) was higher than that of SMZ (0.23X, range, 0.1–0.46X) regardless of the dose [110].

Finally, oral cotrimoxazole 0.16/0.8 g q12h gave **ISF**/plasma ratios of 0.68–1.41X for TMP and 0.39–0.83X for SMZ, with T_max_ values of 2 h for both drugs [159].

## 3. Discussion

The penetration of antimicrobial drugs into tissues ensures the achievement of clinical recovery from infections and, possibly, the eradication of infective foci. As widely described in the literature, patients that are admitted to ICUs have a variety of clinical and pathological conditions (i.e., the presence of comorbidities) that may significantly influence the outcome of chemotherapy. Factors such as organ failure, increased vascular permeability, and renal replacement therapies may alter the pharmacokinetics of antimicrobials up to a threshold that could be associated with a reduced benefit for the patients. Furthermore, the presence of resistant clones and the need to prevent their diffusion are mandatory prerequisites to prescribe effective doses. Those factors justify the use of antimicrobials in regimens that may be considered off-label for the dose (for example, tigecycline) [122], the route, and modalities of administration (i.e., continuous infusions of linezolid) [106]. Furthermore, the knowledge of tissue penetration of antibacterials in ICU patients may guide the choice of the most effective chemotherapy, according to bacterial strain sensitivity and tissue/plasma penetration ratio. Although therapeutic drug monitoring becomes of utmost importance in antimicrobial stewardship protocols, the correlation of concentrations between plasma and peripheral tissue concentrations may vary among patients, so the prediction of tissue levels could be difficult. Based on those premises, the present review focused on the tissue penetration of antimicrobials, which mainly inhibit bacterial protein synthesis and alter DNA structure and activity, in ICU patients.

The low CSF distribution represents a prevalent feature for many antimicrobials, even if there are some exceptions. For example, levofloxacin 0.5 g q12h and metronidazole 0.5 g q8h did achieve effective CSF/plasma ratios of 0.7X [38,135]. Even linezolid has a high (and variable) CSF penetration [22,100,101,102,103].

Among ICU patients, the onset of pneumonia promptly requires effective treatment. Indeed, VAP may be considered one of the most frequent infections that is reported in the ICU, with incidence rates ranging between 5% and 40% and a mortality rate of approximately 10% [160]. Many drugs that were examined in the present review have a high penetration within the LRT. The empirical treatments and antibiogram-based therapies consider fluoroquinolones for their optimal diffusion into the LRT. Ciprofloxacin, levofloxacin, and moxifloxacin distribute into the ELF and achieve PK/PD threshold values [24,42,48]. Interestingly, the ELF/plasma ratios match similar or higher accumulation rates in alveolar cells. Therefore, these concentrative processes support respiratory fluoroquinolones for LTR infections (LRTI). Due to their low hydrophilicity, fluoroquinolones are less sensitive to changes in the volume of distribution. Additionally, the combination of fluoroquinolones plus anti-pseudomonal beta-lactams could be beneficial in reducing the risk of exitus [121].

The treatment of LTRI may also include tetracyclines, macrolides, and linezolid. Excellent diffusion into the ELF of clarithromycin, azithromycin, doxycycline, and tigecycline ensures therapeutic concentrations. Furthermore, doubling the dose of tigecycline may increase the probability of cure rates [122], especially in patients with a high body mass index [161]. A similar strategy has been identified for linezolid because an i.v. bolus of 0.6 g followed by a continuous infusion of 1.2 g/day was associated with an ELF/plasma ratio of 1X [107]. Finally, isoniazid and rifampin penetrate the ELF well and concentrate in alveolar macrophages [150].

Severe infections of the bone and soft tissues can be cured by linezolid thanks to its high penetration regardless of the severity of sepsis [26,112,113]. Levofloxacin highly penetrate the skin, especially in inflamed tissues [57], while tetracyclines diffuse into ISF regardless of the inflammatory status.

Overall, the scientific literature shows how antimicrobials can penetrate within tissues of ICU patients, and that knowledge may guide dosing to achieve therapeutic concentrations. Indeed, respiratory fluoroquinolones, linezolid, macrolides, and tigecycline have better ELF/plasma ratios. Moreover, those drugs accumulate within the AC, hence strongly sustaining their administration in ICU patients with HAP or VAP. On the contrary, the distribution in other organs and tissues is irregular, the pharmacokinetic variability among patients is high, and the number of studies is relatively low. Despite those factors, the administration of off-label regimens may increase the probability of a recovery from the infections.

The paucity of data for drugs in some tissues depends on the problematic collection of samples. Techniques such as mini-BAL (to collect serial samples of ELF) [162] or microdialysis (for ISF harvesting) [163] can solve that issue. In turn, the possibility of a dense sampling allows the investigation of drug penetration in an extended time, which may correspond to the tissue/plasma ratio of AUC values that were calculated between two consecutive doses. That approach is better than a single time point ratio, because several causes (i.e., blood perfusion, the presence of barriers, and physicochemical properties of the drugs) may delay the equilibrium between the tissue and plasma concentrations.

It is worth noting that changes in tissue penetration of antimicrobials can depend on multiple causes. For instance, hemodialytic procedures can augment the clearance of drugs by both the mechanism of drug removal and drug properties [164]. Linezolid is a paradigmatic example because it is a hydrophilic antimicrobial with a low plasma protein binding (31%) [165]. Therefore, dialytic procedures may significantly influence linezolid clearance [166]. Some authors suggested intensive daily dosing (i.e., 0.6 g q8h) [11], but that approach may expose the patients to an increased risk of toxicities. On the other hand, TDM protocols can guide dose individualization of linezolid (and aminoglycosides as well), hence they are considered valuable in ICU settings for standard dosing and continuous infusions [13,167,168]. Despite the availability of immunoassays, the diffusion of drug monitoring services among hospitals is still limited.

Additional causes of altered pharmacokinetics of antibacterial drugs and their tissue penetration rates include the extracorporeal membrane oxygenation (ECMO) procedure. The effects of ECMO on drug diffusion are variable among the antimicrobials and a limited number of clinical trials have investigated those effects [169]. For instance, the recommended ciprofloxacin dose in ICU patients with ECMO did not differ compared to non-ECMO individuals [170]. On the contrary, amikacin and gentamycin doses could be modified in patients that were undergoing ECMO [171,172]. Moreover, burn injuries can cause rapid and massive changes in plasma protein content that gradually generate a state of hypoalbuminemia 2–5 days later [173]. Hypoalbuminemia has severe consequences for the pharmacokinetics of antibacterial drugs [174]. Indeed, at this stage, pharmacokinetic alterations require increased doses. Finally, augmented renal clearance (ARC) is a multifactorial condition that affects approximately 25% of ICU patients [175]. ARC is a severe cause of altered pharmacokinetics of antimicrobials such as aminoglycosides [176]. Therefore, the risk of subtherapeutic concentrations in plasma (and, consequently, in tissues) should be avoided by “maximizing the dose or using prolonged infusions, or making the decision to switch to another agent” [176]. In all of those pathological situations (i.e., alterations in renal function, burn injuries, dialytic procedures, ECMO, etc.), TDM protocols, careful evaluations of patients’ health status, and knowledge of the antimicrobial penetration rates may guide dose optimization in ICU patients [13,177,178].

In conclusion, the knowledge of tissue penetration ratio values retains its importance, especially in ICU settings, where the expected clinical benefit depends on prompt and adequate chemotherapy to treat infections. The choice of drug doses, the administration scheme, and the evaluation of plasma concentrations by TDM protocols are based on that knowledge, which is still in need of further clinical studies.

## 4. Materials and Methods

Tissue penetration of antibacterials in ICU patients was the sole focus of the present review. Indeed, the tissue/plasma ratio may be useful for drug prescribing and forecasting the effect of such treatments in severely ill patients.

ICU physicians selected the panel antimicrobials among those drugs that are commonly prescribed to treat bacterial infections in critically ill patients. Notably, pharmacological agents included fluoroquinolones, aminoglycosides, macrolides, tetracyclines, oxazolidinones, clindamycin, and others (for a complete list, see Section 4.1).

### 4.1. PRISMA Selection of Literature

The PUBMed database was adopted to collect original research articles that were published in peer-reviewed journals between April and June 2022. The search of relevant bibliography was performed by the following keywords organized in 4 main domains:

- Domain 1: patients and ward: critically ill patient(s) OR intensive care unit OR ICU;
- Domain 2: study type: (study OR trial) AND (clinical OR human OR case series OR case report);
- Domain 3: drug list: antimicrobial(s) AND [gentamycin OR amikacin OR tobramycin OR erythromycin OR clarithromycin OR azithromycin OR ciprofloxacin OR levofloxacin OR ofloxacin OR norfloxacin OR moxifloxacin OR doxycycline OR tigecycline OR linezolid OR clindamycin OR metronidazole OR rifampin OR isoniazid OR chloramphenicol OR clotrimoxazole (trimetoprim-sulfametoxazole)];
- Domain 4: tissue distribution: tissue AND [distribution OR penetration OR diffusion OR pharmacokinetic(s)] AND [brain OR cerebrospinal fluid OR (epithelial lining fluid OR ELF) OR lung OR bronchial secretion OR skin OR interstitial fluid OR abdomen OR (peritoneal OR peritoneum) OR urine OR kidney OR liver OR bile OR bone OR synovial OR spleen OR muscle OR (subcutaneous OR subcutis) OR fat OR adipose].

The AND operator was used to combine the 4 domains. The full articles in English that were retrieved during the first round of literature search were managed by Mendeley software together with all those articles that were obtained by a more specific search through the combination of domains 1–3 and distribution in single tissues, organs, and compartments (i.e., CSF, lung, skin). Duplicates were removed from the database, and two independent reviewers (A.Ca. and A.D.P.) selected articles of interest according to the PRISMA 2020 guidelines [179]. The selection was based on the title and abstract if it was informative enough. Further inclusion criteria for selecting articles included the following information: patients’ number, the type of infection, drug dose, route of administration and infusion duration (i.e., bolus, extended, or CI), frequency of dosing, tissue sampling time points, methods for measuring drug concentrations (i.e., chromatographic methods or microbiological assays), and further relevant data (i.e., hemodialytic procedures). Articles were excluded if they presented the following topics: preclinical studies, epidemiology, microbiology, laboratory techniques, clinical use of antibiotics, TDM, and POP/PK in ICU without explicit reference to tissue penetration of drugs. A third reviewer solved the controversies.

Drug penetration into tissues was described as a percentage of the corresponding plasma concentrations. In particular, the tissue/plasma ratio (or tissue/free plasma ratio) was preferentially based on AUC values to exclude possible errors due to the delay (the hysteresis phenomenon) by which drugs pass from plasma to the tissues. The time of sampling was indicated (i.e., 4 h post-dosing) for single concentration values (i.e., C_max_), and the therapeutic regimens (i.e., the dose, the time interval between consecutive doses, and the route of administration) were shown.

## Figures and Tables

**Table 1:** Tissue/plasma ratio values for fluoroquinolones. For each drug, the different daily doses that were administered to ICU patients are listed in the table.

Drugs	Ciprofloxacin	Levofloxacin	Moxifloxacin	Ofloxacin
**Daily doses**	−400 mg q8h	−500 mg q8h −500 mg q12h −500 mg	−400 mg	−400 mg q12h
CNS	1.9X 0.88 ± 0.99X ^A^ (1 h)			0.5X
CSF	<0.1X	0.71X 0.16–0.71X ^B^	0.5–0.8X	0.73–0.76X
Lung	3.1X	0.3–0.7X 0.1–0.8X		
ELF	1.9X	1.12–2X	0.88–6.95X	
Alveolar cells	>10X	18.5X	24.5X	
Bronchial secretions	1.16X	1.55X ^C^	0.80–0.89X 2.07X ^C^	
Bone	0.68–0.75X	0.35X ^D^–0.7X ^E^ (1.5 h) 0.4X	0.4–0.6X 1X ^F^	0.7X
Skin		1.44X		
Fat	1.40X			
**References**	[17,18,19,20,21,22,23]	[19,20,22,24,25,26,27]	[19,22,27,28,29,30]	[31]

**Notes**: ^A^, mean ± standard deviation values; ^B^, minimum-maximum values across the selected references; ^C^, spongious bone; ^D^, cortical bone; ^E^, gut; ^F^, synovial fluid. **Abbreviations**: CNS, central nervous system; CSF, cerebrospinal fluid; ELF, epithelial lining fluid; q8h and q12h, every 8 and 12 h, respectively.

**Table 3:** The tissue/plasma ratio values of linezolid, doxycycline, and tigecycline. For each drug, the different daily doses that were administered to ICU patients are listed in the table.

Drugs	Linezolid	Doxycycline	Tigecycline
Daily doses	−0.6 g q12h	−0.1 g q12h	−0.1 g LD, 0.05 g q12h −100 mg
CNS			0.5X (24 h)
CSF	0.5–0.9X ^A^	0.26X	0.2X
ELF	0.97X (IQR 0.8–1.08X)		1.7X
Bone	0.3–0.7X	0.7X	0.41–2X
Skin	0.75X	0.47X (2 h)	
**References**	[100,101,102,103,104,105,106,107,108,109,110,111,112,113]	[114]	[115,116]

**Notes**: ^A^, minimum-maximum values across the selected references. **Abbreviations**: CNS, central nervous system; CSF, cerebrospinal fluid; ELF, epithelial lining fluid; IQR, interquartile range; q12h, every 12 h.
